# Scleromyxedema and Breast-Implant Associated Lymphoma: Investigating an Unusual Clinical Association

**DOI:** 10.7759/cureus.72933

**Published:** 2024-11-03

**Authors:** Eric Cline, Chase Pitchford, Joshua Brady, Omar Bari, Jeffrey D McBride

**Affiliations:** 1 Department of Dermatology, University of Oklahoma Health Sciences Center, Oklahoma City, USA

**Keywords:** autoimmune skin conditions, breast implant-associated anaplastic large cell lymphoma, case report, dermatology, dermatopathology, lymphoma, oncolology, paraproteinemia, scleromyxedema, spindle cell neoplasms

## Abstract

Scleromyxedema, a rare skin condition, is characterized by a waxy-appearing papular eruption that tends to impact middle-aged adults. Scleromyxedema is often linked to monoclonal gammopathies. However, some patients do not have a coinciding monoclonal gammopathy and experience an atypical presentation of the disease. Rarely have there been reported instances of scleromyxedema related to lymphoma. In this case report, we present a woman being evaluated for breast-implant-associated anaplastic large cell lymphoma (BIA-ALCL) who ultimately received the diagnosis of scleromyxedema.

## Introduction

Scleromyxedema is a rare, chronic, and progressive disorder marked by a cutaneous papular eruption that can progress to sclerodermoid induration. This condition primarily affects middle-aged adults [1]. Affected patients classically develop numerous waxy, firm papules typically involving the hands, forearms, head, neck, upper trunk, and thighs [2]. There is no apparent predilection for race or sex [3]. Scleromyxedema is often associated with immunoglobulin G lambda paraproteinemia and primarily affects the skin, although extracutaneous manifestations have been described, with one case presenting as seizures and acute psychosis [4]. 

The histopathologic features of scleromyxedema include interstitial mucin deposition, dermal fibroblast proliferation, and fibrosis. These histologic changes occur within the dermis, characterized by collagenous and fibroblastic proliferation with variable amounts of dermal mucin. A distinctive feature is the proliferation of spindle cells arranged in fascicles, accompanied by a reduction in the number of elastic fibers. Atrophy of the epidermis and hair follicles is commonly seen. Microscopically, there may be a slight perivascular, superficial lymphocytic, and plasmacytic infiltrate [5].

Breast-implant-associated anaplastic large cell lymphoma (BIA-ALCL) is a rare hematologic malignancy and typically begins as a seroma accompanied by breast swelling or pain. Axillary lymphadenopathy is associated with some cases. Implants can rupture due to the chronic inflammation that occurs in this condition. Textured implants are a greater risk factor for causing this type of lymphoma. The diagnosis is made by imaging and analysis of fluid aspiration [6].

## Case presentation

**Figure 1:**
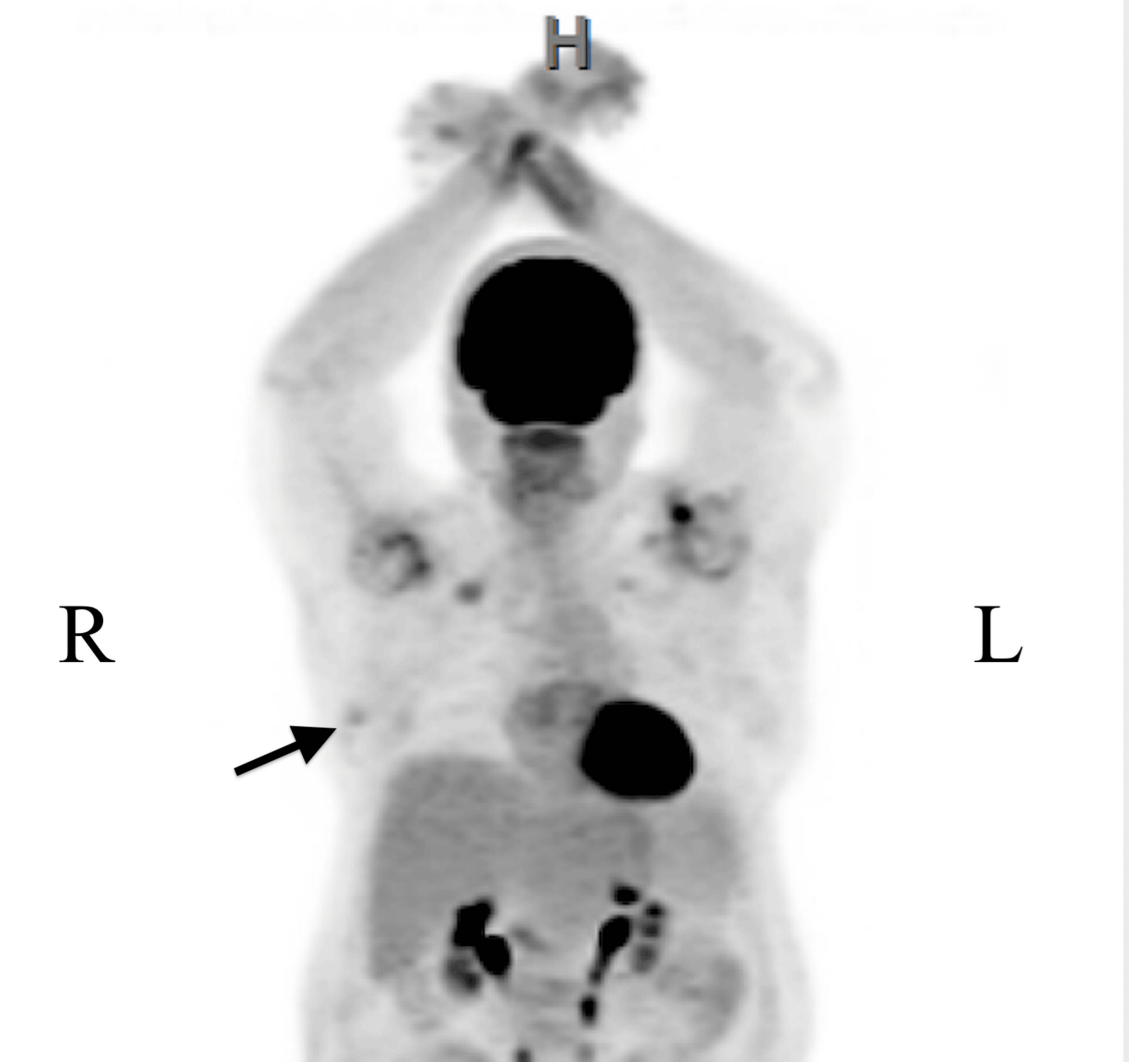
Whole body PET/CT scan with FDG. A mild FDG-avid 1.7 by 0.7 cm right breast nodule concerning for malignancy (black arrow). No focal left breast hypermetabolism is present. FDG: fludeoxyglucose-18.

**Figure 2:**
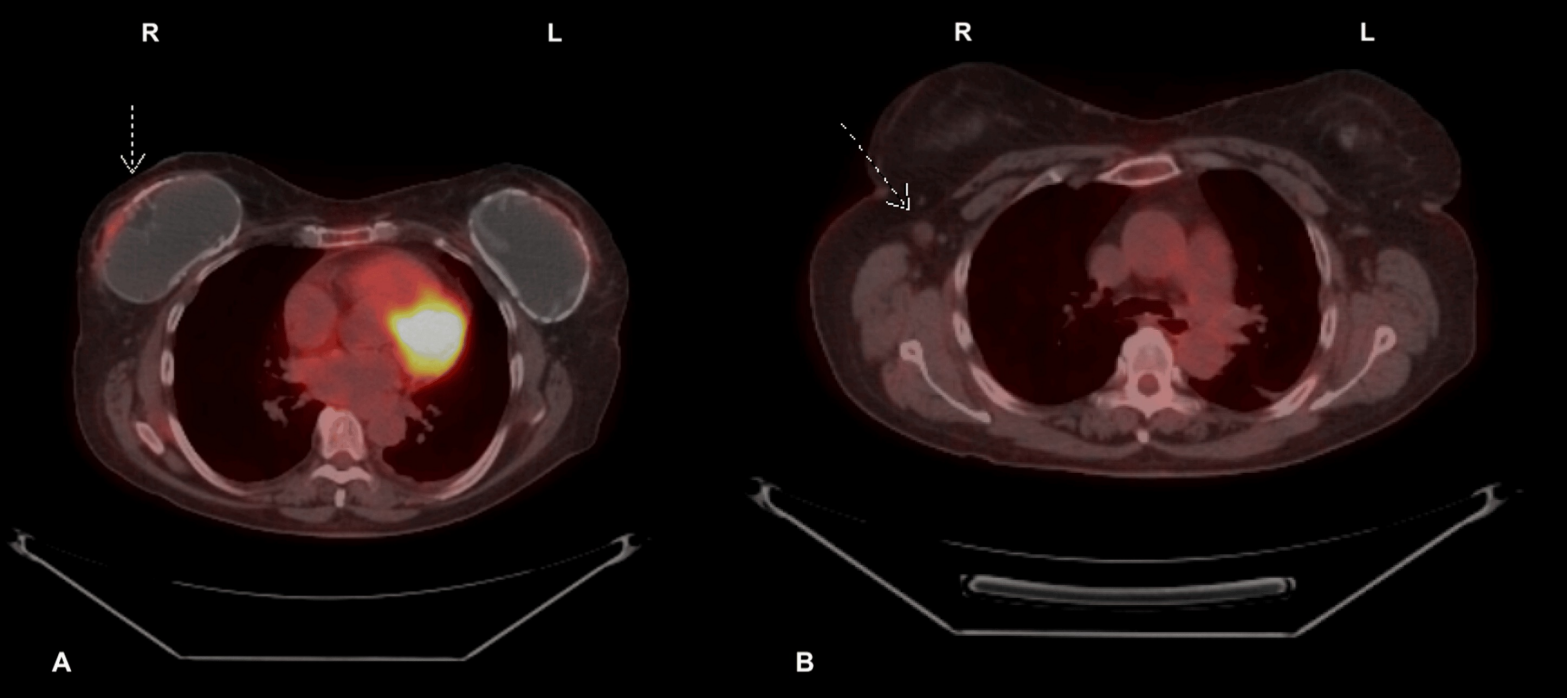
PET/CT scan with FDG; axial view. A: A mild FDG-avid 1.7x0.7 cm right breast nodule concerning malignancy (white arrow). No focal left breast hypermetabolism is present. B: Mildly prominent 1.1x0.9 cm right axillary node (white arrow).

A 64-year-old woman who was being worked up for suspected lymphoma after breast implant rupture came to the hospital for lesions on the hands, elbows, and posterior neck that had progressively worsened for the last two months and were associated with pain and swelling of the hands. Positron emission tomography/computed tomography (PET/CT) scan of the whole body demonstrated a fludeoxyglucose (FDG) avid right breast nodule anterior to the right breast prosthesis concerning malignancy (Figure 1 and Figure 2).

**Figure 3:**
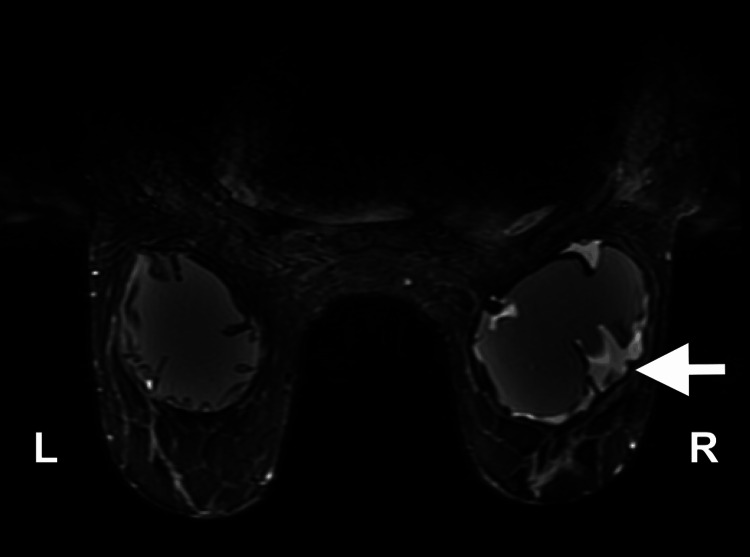
Bilateral MRI of the breasts. Intact right sub-glandular silicone breast implant with a small peri-implant fluid collection with significant mass effect and nodular enhancement along the anterolateral aspect of the implant capsule measuring 3.4x0.8x0.9 cm at the 9 o’clock position (white arrow).

Further MRI of the breasts demonstrated nodular enhancement along the right breast implant, corresponding to the area of increased activity on the prior PET/CT scan, with a small peri-implant fluid collection, raising suspicion for implant-associated lymphoma (Figure 3). 

**Figure 4:**
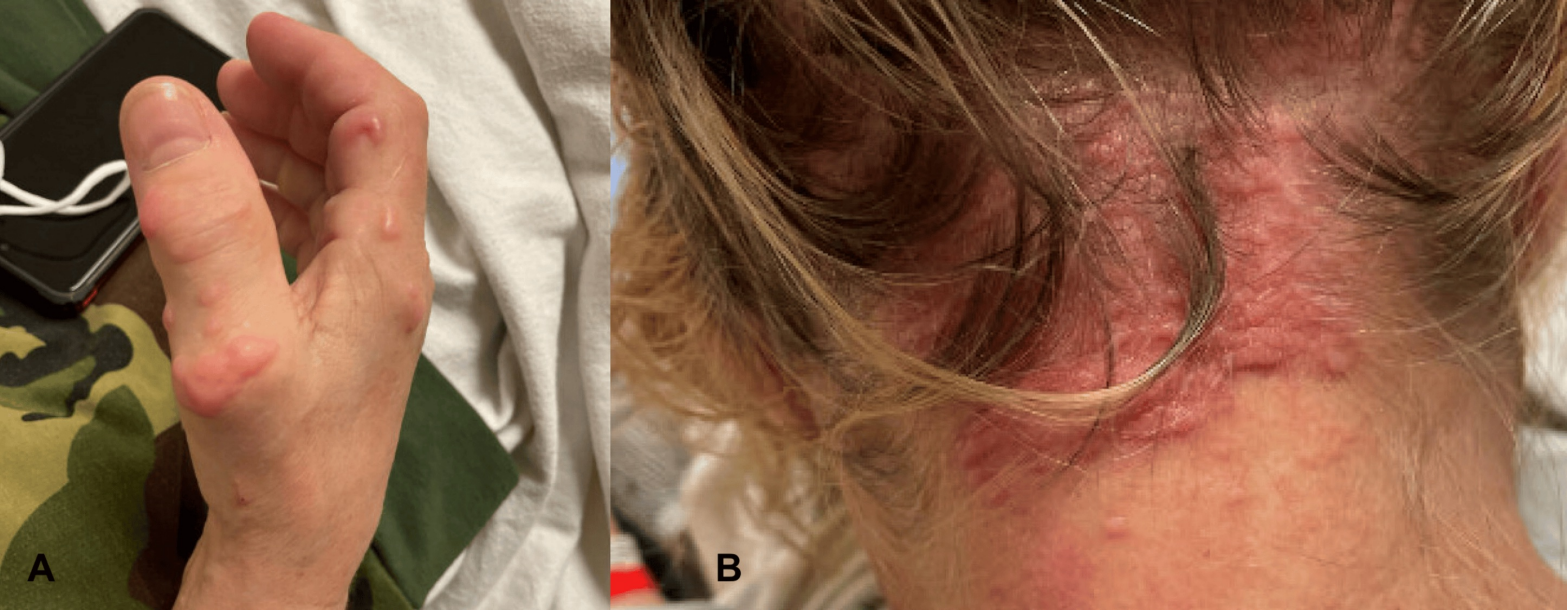
A. right hand; B. posterior neck. A: Pink, shiny, dome-shaped papules and plaques arranged linearly. B: Numerous firm, pink papules coalescing into pebbly plaques.

On physical examination, there were firm, skin-colored to pink papules and nodules on the bilateral dorsal hands and right extensor elbow (Figure 4A). On the posterior neck, there were numerous firm, pink papules coalescing into pebbly plaques (Figure 4B).

**Figure 5:**
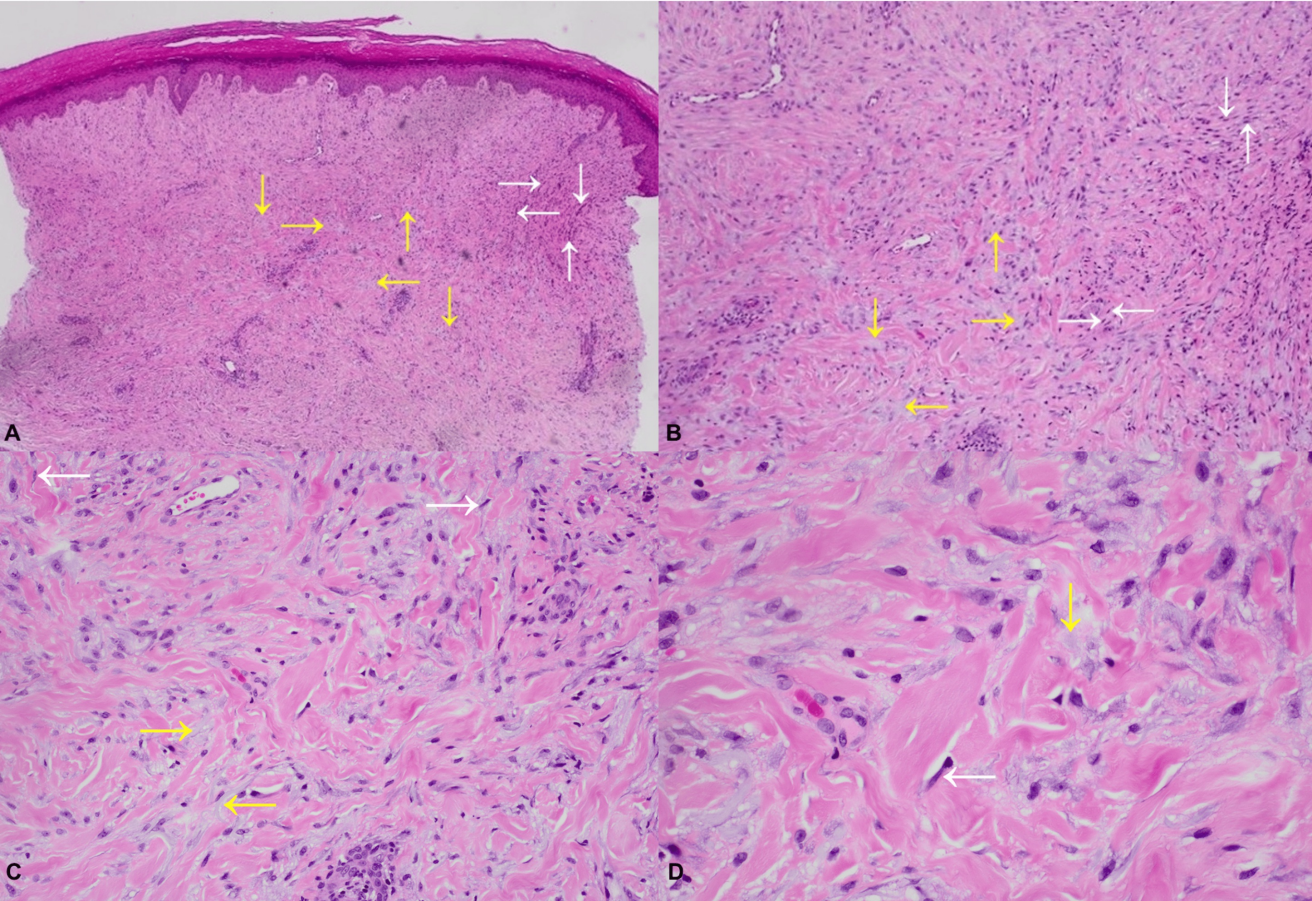
Skin biopsy with hematoxylin and eosin stain. A. Low power view; B. Low power view; C. Medium power view; D. High power view Spindle cell proliferations are variably arranged in fascicles composed of relatively bland, elongated cells (white arrows) with areas of interstitial vacuolated material with a faint blue hue resembling mucin (yellow arrows).

Two punch biopsies, one from the hand and the other from the neck, were collected. Histopathology showed spindle cell proliferations arranged in fascicles with interstitial vacuolated material containing a faint blue hue, representing mucin (Figure 5A, Figure 5B, Figure 5C, and Figure 5D). Spindle cells were negative for SMA, desmin, Sox-10, and CD68 on immunohistochemical stains. Neither eosinophilic amorphous material nor foamy histiocytes were present. This was interpreted as scleromyxedema, and we hypothesize that an undiagnosed lymphoma may have caused this clinical presentation.

An extensive workup by rheumatology found negative rheumatoid factor, cryoglobulins, anti-cyclic citrullinated peptide, serum protein electrophoresis, urine protein electrophoresis, and HIV testing. The patient had been initiated on corticosteroids, which gave her symptomatic relief at a follow-up visit a few months later. She continues to be followed by a multidisciplinary team for continued disease management.

## Discussion

Our case had classic clinical and histopathological findings of scleromyxedema, but the absence of a monoclonal gammopathy, which is seen in 83% of cases, is unusual for this disease process. Additionally, there have been rare associations with hematologic malignancies such as Hodgkin and non-Hodgkin lymphomas and visceral carcinomas [2].

Few studies of high quality on the efficacy of treatments are reported in the scientific literature. Additionally, the pathogenesis of the disorder is not completely understood, which has prevented the development of definitive treatment guidelines for scleromyxedema. Evidence currently does not suggest any specific treatment appears to be universally effective or curative, and the relative efficacies of the treatment options that have been employed in the past remain unclear [3]. Intravenous immunoglobin (IVIG) is considered to be the preferred initial treatment modality based on numerous case studies that support its efficacy. Due to the non-immunosuppressive mechanisms of IVIG, this therapy is generally well-tolerated by patients. However, in those who cannot receive IVIG, systemic glucocorticoids and immunomodulatory drugs, such as lenalidomide or thalidomide, are the preferred first-line systemic treatments [7].

In every case, the risk-to-benefit ratio of treatment is weighed and is important for selecting an appropriate therapeutic regimen [8]. In the rare case of scleromyxedema secondary to a neoplasm, chemotherapy targeted to the primary neoplasm has been shown in a few cases to improve or clear the skin lesions entirely [9]. There is a consensus that a significant improvement in skin thickening and papules is a successful response to therapeutics. However, even with remission after treatment, relapse is possible, necessitating close follow-up with a multidisciplinary team [10].

## Conclusions

This clinical case demonstrates the rarity of lymphoma as the trigger for developing scleromyxedema. The patient is awaiting intervention from a breast surgeon to perform cytology of the breast pocket, which is concerning for BIA-ALCL. Treatment of an underlying malignancy may result in clinical improvement of the patient’s cutaneous findings. There will be continued coordination of care between the dermatology, general surgery, and plastic surgery teams to monitor the patient’s progress. The unforeseeable clinical course of scleromyxedema, varying responses to therapeutics, and potential for relapse demand close, long-term follow-up of patients with this condition.
